# Editorial: Current state and future directions of cranial focused ultrasound therapy

**DOI:** 10.3389/fneur.2022.946634

**Published:** 2022-07-18

**Authors:** J. L. Chazen, Francesco Sammartino, Vibhor Krishna

**Affiliations:** ^1^Department of Radiology, Hospital for Special Surgery, New York, NY, United States; ^2^Department of Neurosurgery, The Ohio State University, Columbus, OH, United States; ^3^Department of Neurosurgery, University of North Carolina, Chapel Hill, NC, United States

**Keywords:** MRgFUS, focused ultrasound (MRgFUS), HIFU, magnetic resonance-guided focused ultrasound surgery (MRgFUS), focused ultrasound (FUS)

Transcranial focused ultrasound (FUS) is a transformative technology to treat neurological and psychiatric disorders. Following pre-clinical development for decades, FUS ablation received FDA approval for essential tremor (ET) treatment in July 2016 (1). The field has undergone rapid development with innovations in image guidance (2, 3), technique optimization (4), and expansion of target selection beyond the thalamus (5–7). In addition to cranial ablation, focused ultrasound can transiently open the transient blood-brain barrier (BBB) in targeted locations and is being actively tested for applications in Alzheimer's disease and brain tumors for targeted drug delivery (8–10).

The current collection of articles highlights the recent innovations in clinical and investigational applications of FUS. Walker et al. discuss the application of DWI and diffusion tractography to visualize the sciatic nerve in piglets They demonstrate reliable sciatic nerve visualization and disruption following MRgFUS ablation in an animal model with histopathologic correlation. This research expands the reach of MRgFUS ablation to the peripheral nervous system and may lead to clinical applications for pain relief with durable nerve conduction blocks (11, 12).

Ahmed A-K et al. explore the impact of ablation location (thalamotomy vs. pallidotomy) on treatment efficiency in a cohort of 40 patients with matched skull density ratios (SDR). Acoustic and thermal simulations were performed at each target, and the findings confirm that globus pallidus interna ablation, located further from the geometric center of the skull, was associated with a higher energy requirement when compared with thalamic ablation. This data has important implications for patient selection for pallidotomy and other off-center ablation targets.

Ahmed N et al. present a thorough review of MRgFUS applications for therapeutic cell delivery in the brain through BBB opening techniques. The authors review the existing methodology of intracerebral cell delivery, including vascular, intrathecal, and stereotactic delivery techniques. Current and future potential for clinical translation is reviewed with applications in neurodegenerative disease, malignancy, autoimmune disorders, and stroke therapy.

Stanziano et al. present data on the resting-state functional MRI connectome in patients with tremor-dominant Parkinson's disease undergoing FUS thalamotomy. Baseline, 1-month, and 3-month connectome data were evaluated for differences in connectivity using a comparative region of interest analysis. The results shed light on changes in functional connectivity between primary motor cortices, supplementary motor cortices, cingulate cortex, and lobe VI of the cerebellar hemispheres. In addition, correlative changes in functional connectivity were observed to a different extent in patients who had a positive clinical response.

Pujol et al. present DTI tractography parcellation of the hyperdirect pathway projections from the M1 cortex and reveals a somatotopic organization by the trunk, arm, hand, face, and tongue. This study analyzed the Human Connectome Project data and should have important implications for patient-specific tractography. By defining patient-specific somatotopy, practitioners could better define ablation targets and avoid off-target effects from FUS ablation.

Agrawal et al. present a systemic review and meta-analysis pooling results from 29 studies of the clinical outcomes and complications of MRgFUS ventral intermediate (Vim) thalamic nucleus ablation for ET patients. Importantly, the analysis revealed a statistically significant reduction in postprocedural ataxia when DTI-based targeted was employed. This review not only supports robust clinical outcomes from Vim ablation in ET patients but reinforces the benefits of diffusion tractography in target selection.

Fishman and Fischell present a review of existing and future techniques for BBB opening to treat neurodegenerative disease. Pre-clinical studies on the delivery of growth factors, antibodies, viral vectors, and nanoparticles into the brain with targeted BBB opening are reviewed. Safety data on the BBB opening technique is presented with a thoughtful reflection on future directions.

Lak et al. present a single-center experience with 160 thalamic FUS ablations in patients with ET or tremor-dominant Parkinson's disease, the largest experience published thus far. They report MRgFUS thalamotomy is a safe and effective procedure with a mean 78% tremor reduction at 2 years. In addition, the most observed side effects at 2 years were imbalance followed by sensory disturbance. This data further bolsters existing literature on the safety and efficacy of MRgFUS thalamotomy.

We anticipate this collection will be of great interest to seasoned and new practitioners of cranial MRgFUS. The field has undergone rapid innovation with exciting future horizons.

## Author contributions

JC, FS, and VK: manuscript preparation and editing. All authors contributed to the article and approved the submitted version.

## Conflict of interest

The authors declare that the research was conducted in the absence of any commercial or financial relationships that could be construed as a potential conflict of interest.

## Publisher's note

All claims expressed in this article are solely those of the authors and do not necessarily represent those of their affiliated organizations, or those of the publisher, the editors and the reviewers. Any product that may be evaluated in this article, or claim that may be made by its manufacturer, is not guaranteed or endorsed by the publisher.
